# Effects of remote coaching following supervised exercise oncology rehabilitation on physical activity levels, physical fitness, and patient-reported outcomes: a randomised controlled trial

**DOI:** 10.1186/s12966-024-01561-2

**Published:** 2024-01-25

**Authors:** Anouk T.R. Weemaes, Milou Beelen, Matty P. Weijenberg, Sander M. J. van Kuijk, Antoine F. Lenssen

**Affiliations:** 1 Department of Physical Therapy, Maastricht University Medical Center+, P.O. Box 5800, Maastricht, AZ 6202 The Netherlands; 2 Department of Epidemiology, Care and Public Health Research Institute (CAPHRI), Faculty of Health Medicine and Life Sciences, Maastricht University, Maastricht, The Netherlands; 3 Department of Human Biology, School of Nutrition and Translational Research in Metabolism (NUTRIM), Faculty of Health Medicine and Life Sciences, Maastricht University, Maastricht, The Netherlands; 4 Department of Epidemiology, GROW School for Oncology and Reproduction, Faculty of Health Medicine and Life Sciences, Maastricht University, Maastricht, The Netherlands; 5 Department of Clinical Epidemiology and Medical Technology Assessment, Maastricht University Medical Center, Maastricht, The Netherlands

**Keywords:** Tele rehabilitation, Accelerometry, Physical activity maintenance, Behaviour change

## Abstract

**Background:**

Studies have shown that cancer survivors experience difficulties maintaining physical activity levels after participation in a supervised exercise rehabilitation program. This study aimed to assess the effectiveness of a six-month remote coaching intervention, following a supervised exercise oncology rehabilitation program on maintenance of PA levels; and improvement of aerobic capacity, muscle strength and patient-reported outcomes in cancer survivors.

**Methods:**

Ninety-seven participants from a Dutch University Hospital’s exercise rehabilitation program were randomised to the COACH group (*n* = 46), receiving 6 months of remote coaching after completing the exercise program, or the CONTROL group (*n* = 50), receiving no additional intervention. Assessment of PA levels; sedentary time; aerobic capacity; muscle strength; fatigue; health-related quality of life (HRQoL); level of anxiety and depression; and return to work (RTW) rates were conducted at baseline (T0) and six months later (T1). Multiple linear regression was used for between-group statistical comparisons of all outcomes measures. Mean differences at T1 were estimated with corresponding 95% confidence intervals (95%CI).

**Results:**

No significant between-group differences were observed for all outcomes at T1. An adjusted mean difference in weekly PA of 45 min (95%CI -50;140) was observed between the COACH group and the CONTROL group, favouring the COACH group, yet lacking statistical or clinical significance.

**Conclusions:**

Our six-month remote coaching intervention did not notably improve PA levels; sedentary time; aerobic capacity; muscle strength; HRQoL; fatigue; anxiety and depression symptoms and RTW rates after participation in a supervised exercise oncology program. Although the participants who received coaching showed slightly higher levels of PA, these differences were not significant. More research is needed to identify patients in need for follow-up interventions following supervised exercise program and to investigate the effectiveness of remote coaching interventions in these patients.

**Trial registration:**

Dutch Trial Register NL7729, registered 13 may 2019, https://trialsearch.who.int/Trial2.aspx?TrialID=NL7729.

**Supplementary Information:**

The online version contains supplementary material available at 10.1186/s12966-024-01561-2.

## Background

Cancer survivors often experience a variety of physical and psychosocial complaints, such as decreased aerobic capacity and muscle strength, fatigue, and symptoms of anxiety and depression [1]. These issues can persist for many years after completing medical treatment and can result in chronic fatigue, decreased physical activity (PA) levels, difficulties to return to work (RTW), impaired social involvement, and consequently a diminished health-related quality of life (HRQoL) [2, 3]. For cancer survivors, participating in an exercise rehabilitation program is a way to increase their PA levels.

Although positive short-term effects of exercise on physical and psychosocial complaints in cancer survivors have been described extensively, few studies report on long-term effects and PA maintenance after completing a supervised exercise program [1, 4, 5]. Kampshoff et al. reported that improvements in aerobic capacity and HRQoL persisted until 64 weeks after completing an exercise intervention in patients with different types of cancer, while fatigue returned to baseline level. Moreover it turned out that levels of aerobic capacity were still ‘poor’ when compared to healthy adults [6]. To further improve the health benefits that are achieved during an exercise program, patients have to stay physically active. However, it seems challenging for cancer survivors to sustain PA levels after completing a supervised exercise program. The literature indicates that short-term supervised exercise programs may be insufficient for cancer survivors to reach and sustain PA levels that meet current guidelines [5–8]. Results of a qualitative study suggested that cancer survivors experience the transition from a supervised hospital-based exercise program to independent community-based exercise as difficult. This transition could be improved through a more structured transition, accessibility of transferable tools, sustained peer support and ongoing monitoring [8]. 

In recent randomised studies, it was shown that remote interventions, like text messages and health coaching delivered during and after a structured exercise program, can promote PA maintenance in cancer survivors [9–11]. However, in two of these studies [9, 10], interventions lasted only for 8 weeks, which may be too short for habit formation [12], and long-term effects were not assessed. Besides, the effects of remote coaching on physical and psychosocial complaints were not examined in these previous studies. Therefore, the aim of this study was to examine the efficacy of a six-month remote coaching intervention, delivered after a supervised exercise program, on maintenance of PA levels; and on improvement of aerobic capacity; muscle strength; HRQoL; fatigue; anxiety and depression symptoms and RTW rates in cancer survivors.

## Methods

### Design

This single-blind randomised controlled trial (RCT) recruited participants between May 2019 and December 2021, from a usual care, supervised 10-week exercise program which was part of usual care multidisciplinary oncology rehabilitation at the Department of Physical Therapy of the Maastricht University Medical Centre (MUMC+) in the Netherlands. Patients were screened for eligibility and asked to participate during the last week of the exercise program. The content of this exercise program as part of multidisciplinary rehabilitation that was aimed at improving aerobic capacity and muscle strength has been described elsewhere [13]. Patients who were willing to participate, gave written informed consent. After baseline measurements, participants were randomised either to the intervention group (COACH) or the control group (CONTROL) in a 1:1 ratio. The allocation sequence was generated by an independent researcher using a computer-based random number generator and was stratified for age (≤ 55 or > 55 years old) and sex in blocks of four. The allocation sequence was concealed for the researcher who enrolled participants and assigned them to groups, using sequentially numbered, sealed envelopes. Procedures of data collection were in compliance with the Declaration of Helsinki and were approved by the Medical Ethics Committee of MUMC + with registration number 18–050. The study is reported according to the Consolidated Standards of Reporting Trials (CONSORT) guidelines [additional file 1] and was registered as NL7729 in the Dutch Trial Register (https://trialsearch.who.int/).

### Participants

Patients were eligible to participate in this study when they were ≥ 18 years old; were suffering from physical, and/or psychosocial complaints and/or chronic fatigue; and completed active medical treatment (i.e. surgery, chemotherapy, radiotherapy, stem cell transplantation) and a 10-week exercise program, as part of multidisciplinary oncology rehabilitation. Patients were excluded if they had insufficient understanding of the Dutch language, were in an unstable phase of disease (e.g. receiving palliative treatment), scheduled for chemotherapy, radiation or invasive surgery in the next six months and if they were unable to perform exercise activities without supervision (i.e. because of risk of falling or injuring).

### Intervention

The six-month remote coaching intervention was delivered by a community-based sports organisation (Maastricht Sport, Municipality of Maastricht, The Netherlands) and aimed to stimulate patients to increase their PA levels. This intervention is already successfully implemented in usual care for patients who completed an exercise cardiac rehabilitation program at the MUMC+. Involved coaches had at least a bachelor’s degree in Sport Science or Sports and Movement Education, were trained in behaviour change techniques and were experienced in delivering the intervention. During a face-to-face intake assessment at the Department of Physical Therapy of the MUMC+, the coach obtained information about the subjects’ personal motivation and PA preferences, using the Capability, Opportunity, and Motivation model of Behaviour (COM-B model). In this model about behaviour change, capability (physical and psychological), opportunity (physical and social) and motivation (automatic and reflective) are seen as the drivers of behaviour [14]. The coaches identified facilitators and barriers for behaviour change in these three constructs using a self-developed questionnaire and adapted the coaching accordingly. The questionnaire is reported in an additional file [additional file 2], with the percentage of participants who answered ‘yes’ and ‘no’. After the intake, the program consisted of individually tailored, remote coaching. The coaching took place via phone calls or e-mails, depending on personal preferences. In the first three months, the coach approached the subjects weekly. Thereafter, the coach evaluated the individual progress and the frequency was reduced to one contact moment per month. Attendance to the intervention was reported by the coach and adherence (%) was calculated by the researcher at the end of the study. In case of e-mail contact, participants had to respond by sending a reply e-mail to adhere to the intervention. The intervention was reported according to the Template for Intervention Description and Replication (TIDieR) guidelines [additional file 3].

### Control group

The control group received no additional intervention. However, during the prior rehabilitation program, all patients were encouraged to reach PA levels that meet the World Health Organization (WHO) guidelines and the healthcare providers advised all patients to sustain these PA levels and informed them about possibilities for suitable community-based exercise in their neighborhood.

### Measurement procedures

Due to the nature of the intervention, it was impossible to blind participants and care providers. However, the researcher who performed data collection and data analysis was blinded until after data analysis, and validated, objective measurement tools were used in order to minimise risk of bias. Measurements of accelerometer-derived and patient-reported PA levels, aerobic capacity, muscle strength, fatigue, HRQoL, anxiety and depression and RTW rates were performed during the last week of the exercise rehabilitation program (T0) and were repeated six months later (T1). Patient characteristics were obtained from medical records. Self-reported PA levels before diagnosis were assessed at baseline, during short structured interviews. During this interview, participants reported the number of hours per week they walked, cycled or performed any other kind of exercise before the diagnosis.

***Accelerometer-derived PA levels*** were assessed using the validated, waterproof, thigh-mounted tri-axial MOX accelerometer (MMOXX1; Maastricht Instruments B.V.; Maastricht; the Netherlands [15, 16]. The MOX showed good test-retest reproducibility (kappa 0.95) and good validity compared to direct observations (kappa 0.99) for differentiating between postures (lysing down/sitting and standing) and PA in a laboratory setting. Besides, the MOX has good validity for estimating time spent in the same categories in free-living conditions, compared with diary records (intraclass correlation coefficient (ICC) 0.98) [17]. The MOX accelerometer was attached to the right upper thigh, 10 cm proximal of the patella using a non-allergic plaster. Subjects wore the accelerometers 24 h/day during 7 consecutive days. With embedded software, acceleration was converted to counts per second and time could be classified as sedentary (lying down/sitting), standing or PA time. The primary outcome measure of this study was weekly accelerometer-derived total PA time in minutes. Weekly PA time and sedentary time were also calculated as a percentage of waking time.

***Patient-reported moderate-to-vigorous intensity PA (MVPA) levels*** were monitored during the 7-day wear-time of the MOX accelerometer. Subjects were asked to report daily activities spent in MVPA of ≥ 10 min and wake/sleep time in a PA diary. To instruct participants, MVPA was defined as ‘physical activities while standing or moving that increase the breath and heart rate (like brisk walking, cycling, gardening and exercising)’. Activities that were written down were analysed afterwards by the researcher using the compendium of PA and the total number of minutes spent in MVPA (≥ 3.0 metabolic equivalent of task, MET) was calculated [18]. At T1, any consultations with a physical therapist were extracted from the diaries as well, to check for equal distribution of co-interventions between the groups.

***Aerobic capacity*** was examined during a maximal incremental exercise test with respiratory gas analysis, usually referred to as the cardiopulmonary exercise test (CPET). Measuring the highest amount of oxygen consumed during peak exercise (VO_2_peak) during CPET is the criterion standard to evaluate aerobic capacity, has sufficient test-retest reproducibility (coefficient of variation 6%) [19] and is safe and feasible in patients with cancer [20]. Height and weight was measured prior to the test. The CPET was performed on an electronically braked cycle ergometer (Lode Corival; Lode BV, Groningen, The Netherlands). The test consisted of a two-minute rest period, a three-minute warm-up phase of unloaded cycling and a test-phase with an incremental ramp-protocol, adjusted to the patient’s self-reported PA level, aimed at reaching a maximal effort within eight to twelve minutes. Continuous breath-by-breath analysis was obtained throughout all the phases of the test using an ergospirometry system calibrated for respiratory gas analysis measurements and volume measurements (Vyntus CPX, CareFusion, the Netherlands). Participants were instructed to keep cycling until exhaustion, with a pedaling frequency of at least 60 rotations per minute (rpm). The protocol continued increasing until the patient stopped cycling or pedaling frequency fell below 60 rpm, despite strong verbal encouragement. Voluntary exhaustion was considered to be achieved when participants showed clinical signs of intense effort (e.g., unsteady biking, sweating or clear unwillingness to continue exercising). CPET results were analysed by a trained researcher who was blinded for group allocation and moment of testing (T0 or T1), using a standardized protocol. Values of oxygen uptake (VO_2_) and the respiratory exchange rate at peak exercise (VO_2_peak and RER-peak, respectively) were averaged over 30s. VO_2_peak values were also converted to percentages of reference values for the Dutch general population and the number of participants that reached a VO_2_peak beneath the lower limit of normal was reported [21]. The following submaximal CPET outcomes were determined as well, as described elsewhere: VO_2_ at the ventilatory anaerobic threshold (VO_2_VAT), VO_2_ at the respiratory compensation point (VO_2_RCP) and the oxygen uptake efficiency slope (OUES) [13]. 

***Muscle strength*** of the lower and upper extremity was measured during submaximal repetition maximum (RM) tests on the leg press and chest press machine. An indirect determination was used, because performing a direct 1-RM test is not feasible in patients and could cause injuries. The indirect RM test was performed with a weight that allowed for a maximum of 5 repetitions. This weight was estimated and the participants were asked to perform the maximum achievable number of repetitions up to 5 repetitions. When more than 5 repetitions could be reached, the weight was increased and participants repeated the exercise after a 1-min break until they no longer reached > 5 repetitions. True 1-RM values were calculated afterwards using the Brzycki equation [22]. The indirect RM-test was found to have a good test-retest reproducibility in untrained persons (ICC > 0.99) [23]. 

***Health-related quality of life*** was measured using the European Organization for Research and Treatment of Cancer Quality of Life Questionnaire Core-30 (EORTC QLQ-C30). This is a widely used questionnaires to assess HRQoL in patients with cancer, showing good psychometric properties [24, 25]. In this questionnaire, each of the 30 items has to be rated on a scale from 1 to 4 and for two items from 1 to 7. The EORTC QLQ-C30 distinguishes 15 sub-scales. The functioning scales (physical, role, emotional, social and cognitive functioning), the global QoL scale and a functioning sum score (averaged across the 15 items that belong to the functioning scales) were calculated and linearly transformed on a 100-point scale. For these sub scores, higher scores indicate higher levels of HRQoL [26]. 

***Fatigue*** was assessed using the Multidimensional Fatigue Inventory-20 (MFI-20), which is a validated 20-item questionnaire designed to assess fatigue in patients with cancer, using a five-dimensional structure (general, physical and mental fatigue, reduced motivation and activity). Each items is scored on a five-point Likert-scale. The sub scores range from 4 to 20, with lower scores indicating lower levels of fatigue. The sum score was calculated by adding up the sub scores [27, 28]. 

***Anxiety and depression*** was assessed using the validated 14-item Hospital Anxiety and Depression Scale (HADS). Items are scored on a 4-point scale and sub scores for anxiety and for depression range from 0 to 21, with lower scores indicating lower levels of anxiety and depression. The sum score was calculated by adding up the sub scores [29]. 

***Return to work*** was assessed during a short, structured interview. Subjects were asked whether or not they were employed before the diagnosis and for how many hours, if they have reintegrated to the work process and for how many hours/week they were working at the moment of the interview. Return to work was reported as a percentage (%) of pre-diagnosis hours of work per week.

### Sample size calculation

The sample size was calculated a priori in order to be able to identify a clinically relevant difference in mean total PA time between the intervention group and the control group. A sample size which provided sufficient power (i.e. 80%) to detect a clinically relevant difference of 15 min/day or 105 min/week (associated with a 4% reduction in all-cause mortality) [30] between both groups, was pursued. When using the standard deviation (sd) of PA data from a sample of comparable patients (sd = 172.46) [31], a clinically relevant change of 105 min/week, and an α of 0.05 resulted in a total sample size of *n* = 86. Accounting for an expected loss-to-follow up of 10%, we aimed to include 96 patients.

### Statistical analysis

Statistical analyses were performed using SPSS version 28.0 (IBM Corp. Released 2020. IBM SPSS Statistics for Windows. Armonk, NY: IBM Corp). Continuous variables were checked for normality using histograms and Q-Q plots and were presented as mean ± standard deviation (sd) or as median and 1st and 3rd quartile for continuous variables, as appropriate. Categorical variables were reported as frequencies and percentages. Multiple imputation with fully conditional specification was used to impute incomplete records, to minimise potential bias from using complete cases only. The number of imputations was set to fifty, and predictive mean matching was used to draw values to be imputed. Results from inferential statistics were pooled using Rubin’s rules.

Data were analysed on an intention-to-treat basis. Outcomes are reported for measurements at T0 and T1 for both groups, with mean changes from T0 to T1 and corresponding 95% confidence intervals (95% CI). Between-group differences were calculated and reported as appropriate. Multiple linear regression was used for between-group statistical comparisons of all outcomes measures. Adjusted mean differences at T1 were estimated with corresponding 95%CI. Randomisation stratification factors (age and sex) were entered in the regression models [32]. In case of perceived group differences in baseline variables, these variables were entered in the regression model as well. Effect sizes (Cohen’s *d*) of the corrected mean differences were calculated as well. Furthermore, the number of participants that showed a clinically relevant increase (≥ 105 min), remained stable (-105 min–105 min) or showed a clinically relevant decrease (≤ 105 min) in weekly, accelerometer-derived total PA were reported for each group and a Pearson’s chi-square test was used for between-group comparisons.

## Results

### Participants

**Fig. 1:**
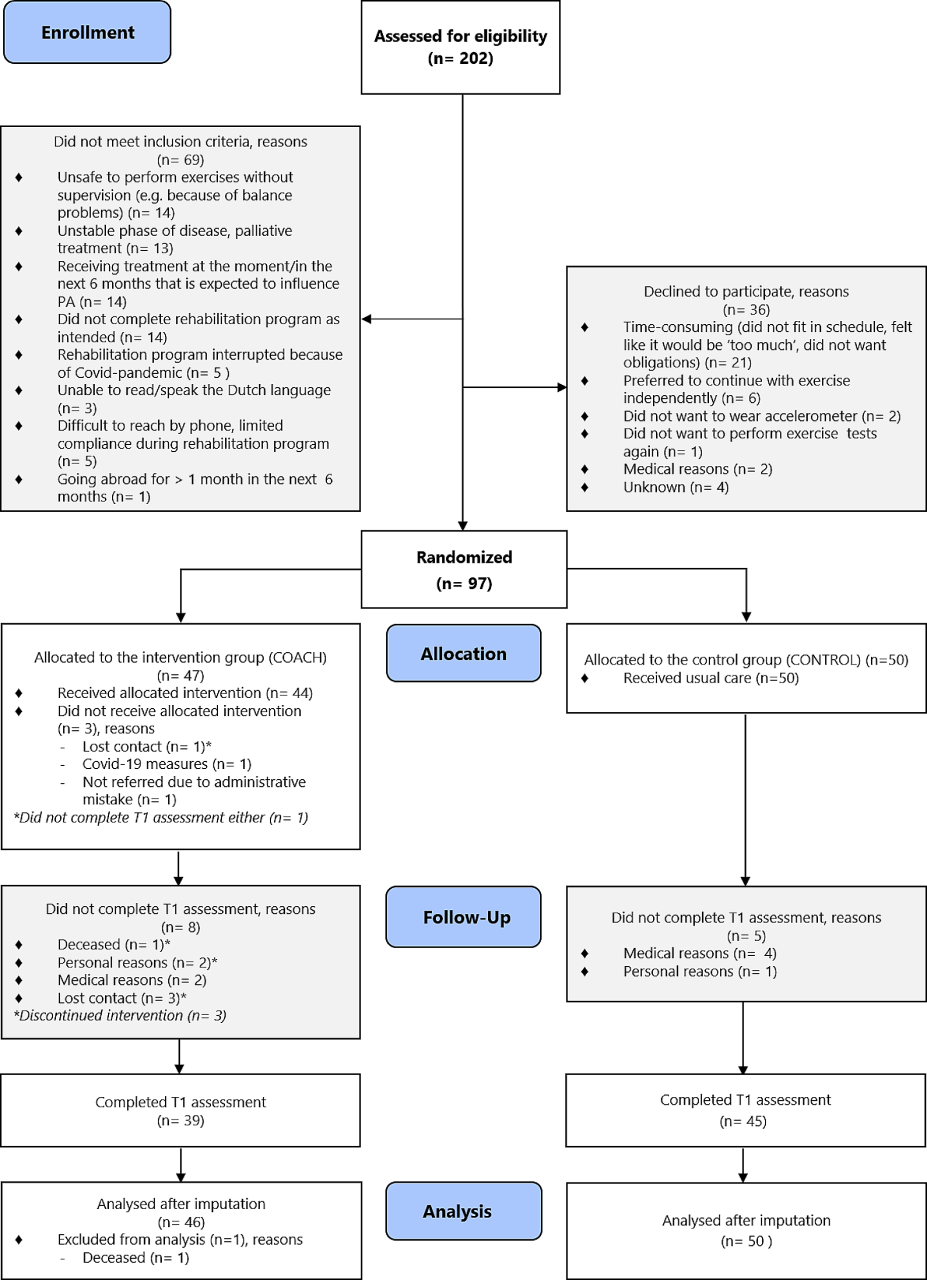
Participant flowchart. Legends: T1 = outcome assessment, 6 months after the start of the study. Covid-19 = Coronavirus-19

Between May 2019 and December 2021, 202 patients participating in the multidisciplinary rehabilitation program of the MUMC + were screened for eligibility. Sixty-nine patients did not meet the inclusion criteria and 36 patients declined to participate. Reasons for exclusion and declining to participate are described in Fig. 1. Ultimately, a total of 97 participants (48%) were included and randomly assigned to the intervention group (COACH, *n* = 47) or the control group (CONTROL, *n* = 50). One participants in the COACH group deceased during the course of the study and was therefore excluded from analysis. (Fig. 1).

Participants who received the intervention (*n* = 43), completed on average 12 of the 15 intended remote coaching appointments, resulting in a mean adherence rate of 83%. Due to the measures during the coronavirus-19 (COVID-19) pandemic, the intake assessments originally scheduled for face-to-face appointments were conducted via phone calls for four participants. After the intake, 7 participants chose to receive the coaching by e-mail, 34 participants received phone calls and 2 participants got a combination of phone calls and e-mails. The duration of the phone calls ranged from 10 to 20 min.

Outcome measures at T1 could not be collected in 8 participants in the COACH group (17%) and 5 participants in the CONTROL group (10%). Medical issues were the most common reason (46%) for drop-out. Reasons for drop-out are described in Fig. 1. For participants who completed the outcome measurements, mean time between T0 and T1 was 27 ± 2.3 weeks in the COACH group and 27 ± 5.8 weeks in the CONTROL group. Missing outcome variables were imputed before further analysis.

Baseline characteristics are presented in Table 1. Breast cancer was the most common diagnosis (55%), the mean age was 54 ± 12 years and mean BMI was 27.5 ± 4.7 kg/m^2^. Based on qualitative appraisal of the baseline characteristics, baseline accelerometer-derived weekly PA differed between the COACH and the CONTROL group. Mean weekly PA was 848 ± 256 min in the COACH group and 894 ± 256 min in the CONTROL group. Other baseline variables were balanced between both groups. (Table 1) Therefore, baseline weekly PA was entered in the regression model as covariate to adjust between-group analyses.

**Table 1:** Baseline characteristics for the COACH group and the CONTROL group

	COACH group *N* = 46	CONTROL group *N* = 50
**Sex (n,%)**		
Male Female	9 (20%) 37 (80%)	12 (24%) 38 (76%)
**Age (years)**	52.9 ± 10.4	55.3 ± 12.5
**Body mass index (kg/m** ^**2**^ **)**	28.0 ± 5.0	26.6 ± 4.2
**Cancer type (n,%)**		
Breast cancer Lung cancer Leukemia Lymphomas Colorectal cancer Head- and neck cancer Other	27 (59%) 2 (4%) 2 (4%) 4 (9%) 3 (7%) - 8 (17%)	26 (52%) 5 (10%) 2 (4%) 1 (2%) 1 (2%) 3 (6%) 12 (24%)
**Metastasis (n, %)**		
Lymphatic metastasis Distant metastasis No metastasis	7 (16%) 3 (7%) 36 (78%)	17 (34%) 1 (2%) 32 (64%)
**Treatment (n,%)**		
Surgery Chemotherapy Radiotherapy Hormone therapy Immunotherapy Stem cell transplantation	38 (83%) 29 (63%) 23 (50%) 15 (33%) 8 (17%) 2 (4%)	41 (82%) 29 (58%) 28 (56%) 16 (32%) 7 (14%) 1 (2%)
**Time since active medical treatment (months)**	7.5 ± 6.1	6.3 ± 4.0
**Comorbidity (n,%)**		
Cardiovascular Respiratory Musculoskeletal Psychological	10 (22%) 1 (2%) 11 (24%) 4 (9%)	11 (22%) 5 (10%) 19 (38%) 7 (14%)
**Self-reported exercise history before diagnosis (hours/week)**	5 ± 5	6 ± 7
**Employed before diagnose**	38 (83%)	39 (78%)
**Weekly physical activity T0 (min)** ^**A**^	848 ± 256	894 ± 256
**Peak oxygen uptake T0 (mL/kg/min)**	22.3 ± 6.1	22.7 ± 6.1
**Quality of life (EORTC-QLQ-C30 sum score)**	73.1 ± 15.6	74.6 ± 15.3
**Fatigue (MFI-20 sum score)**	57 ± 15	54 ± 18
Anxiety and Depression (HADS sum score)	11 ± 6	12 ± 8

Values are presented as n(%) for categorical variables and as mean ± SD for continuous variables

^A^ Accelerometer-derived total physical activity (including physical activity of all intensities e.g. light, moderate and vigorous intensity)

### Within-group changes

At T1, mean weekly accelerometer-derived total PA increased with + 33 min (95% CI -48; 113) in the COACH group and decreased with − 30 min (95% CI -96; 36) in the CONTROL group compared to levels at T0. Both within-group changes were not significant. Besides, the weekly time that participants were sedentary during waking hours decreased with − 147 min (95% CI -396;102) in the COACH group and increased with + 62 min (95% CI -194;317) in the CONTROL group, although not significant. No significant changes over time from T0 to T1 were seen either for mean values of weekly self-reported MVPA, CPET outcomes, upper and lower body muscle strength and different domains and sum scores of HRQoL, fatigue and anxiety and depression, in both groups. RTW increased significantly in both groups, with 29% (95%CI 16;42) in the COACH group and 35% (95% CI 18;51) in the CONTROL group. (Tables 2 and 3)

**Table 2:** Physical activity levels and performance outcomes at T0 and T1 with corresponding changes in both groups

	COACH T0	COACH T1	COACH ∆ (95% CI)	CONTROL T0	CONTROL T1	CONTROL∆ (95% CI)
**Weekly PA level**						
Weekly accelerometer-derived total PA(min) Weekly wake time (min) PA as percentage of waking time (%) Weekly accelerometer-derived sedentary wake time (min)^A^ Sedentary wake time as percentage of waking time (%) Self-reported weekly MVPA (min)	848 ± 256 6332 ± 302 13 ± 4 4157 ± 615 66 ± 9 591 ± 364	881 ± 268 6265 ± 555 14 ± 4 4010 ± 819 64 ± 11 557 ± 400	33 (-48;113) -67 (-279;146) 1 (-1;2) -147 (-396;102) -2 (-5;1) -35 (-189;119)	894 ± 256 6308 ± 280 14 ± 4 3940 ± 639 62 ± 9 619 ± 324	864 ± 253 6298 ± 459 14 ± 4 4002 ± 765 63 ± 11 589 ± 414	-30 (-96;36) -9 (-206;187) 0 (-2;0) 62 (-194;317) 1 (-2;4) -30 (-171;110)
**CPET outcomes**						
VO_2_peak (mL/kg/min) % predicted RERpeak VO_2_VAT (mL/kg/min) VO_2_RCP (mL/kg/min)^B^ OUES	22.3 ± 6.1 71 ± 17 1.18 ± 0.09 13.5 ± 3.3 20.5 ± 5.9 24.8 ± 6.8	22.2 ± 5.9 71 ± 17 1.18 ± 0.08 13.0 ± 4.4 20.3 ± 5.7 24.6 ± 6.5	-0.1 (-1.2;1.1) 0 (-4;4) 0.00 (-0.02;0.03) -0.5 (-2.0;0.9) 0.0 (-1.4;1.5) -0.2 (-1.6;1.2)	22.7 ± 6.1 75 ± 17 1.19 ± 0.10 13.7 ± 3.5 21.7 ± 5.9 25.3 ± 6.1	22.6 ± 6.5 74 ± 18 1.17 ± 0.09 13.2 ± 4.3 20.7 ± 5.9 25.4 ± 6.9	-0.1 (-1.2;1.0) 0 (-4;3) -0.01(-0.04;0.01) -0.5 (-1.7;0.8) -0.4 (-1.7;0.9) 0.2 (-1.1;1.4)
**Muscle Strength**						
1-RM leg press (kg) 1-RM chest press (kg)	135 ± 36 34 ± 13	133 ± 35 34 ± 13	-2 (-11;8) 0 (-3;3)	132 ± 34 34 ± 13	130 ± 35 34 ± 13	-3 (-12;7) 1 (-3;2)

Means ± SD are presented for both groups and timepoints. Mean changes over time (∆) are presented wit corresponding 95% confidence intervals (95% CI). Changes were not statistically significant

^A^ Weekly time that participants were sedentary during waking hours

^B^ The RCP was not always reached during the CPET tests and was not imputed in cases a test was completed correctly and RCP was not reached Group A T0: *n* = 41, T1 *n* = 41; Group B T0: *n* = 37, T1 *n* = 44

COACH = the group of participants receiving a remote coaching intervention; CONTROL = the group of participants receiving no intervention; T0 = baseline; T1 = follow-up

PA = physical activity, MVPA = moderate-to-vigorous physical activity, VO_2_peak = peak oxygen uptake, RER = respiratory exchange rate, VO_2_VAT = oxygen uptake at ventilatory anaerobic threshold, VO_2_RCP = oxygen uptake at respiratory compensation point, OUES = oxygen uptake efficiency slope, 1-RM = one-repetition maximum

**Table 3:** Patient-reported outcomes at T0 and T1 with corresponding changes in both groups

	COACH T0	COACH T1	COACH ∆ (95% CI)	CONTROL T0	CONTROL T1	CONTROL∆ (95% CI)
**Quality of Life (EORTC-QLQ- C30)**						
Global quality of life Physical functioning Role functioning Emotional functioning Cognitive functioning Social functioning Sum score	69.6 ± 17.6 83.1 ± 12.1 68.4 ± 25.6 73.4 ± 21.9 68.9 ± 25.2 75.3 ± 27.8 73.1 ± 15.6	71.7 ± 17.9 84.3 ± 14.8 72.8 ± 26.1 80.6 ± 17.5 75.4 ± 19.9 79.6 ± 26.1 77.4 ± 14.4	2.1 (-4.1;8.3) 1.2 (-3.2;5.5) 4.4 (-5.4;14.2) 7.2 (-0.8;15.2) 6.5 (-0.7;13.7) 4.3 (-5.4;14.1) 4.3 (-0.5;9.1)	69.3 ± 15.1 84.7 ± 12.9 72.7 ± 21.1 74.1 ± 23.1 69.5 ± 25.0 77.5 ± 24.7 74.6 ± 15.3	70.8 ± 19.7 84.1 ± 15.9 71.4 ± 22.4 70.6 ± 25.1 71.3 ± 23.7 75.5 ± 25.3 74.0 ± 16.5	1.6 (-2.6;5.8) -0.6 (-3.9;2.7) -1.3 (-8.4;5.9) -3.4 (-9.8;3.0) 1.8 (-3.8;7.3) -2.0 (-10.6;6.5) -0.7 (-4.4;3.1)
**Fatigue (MFI-20)**						
General fatigue Physical fatigue Reduced motivation Reduced activity Mental fatigue Sum score	13 ± 4 11 ± 4 10 ± 4 11 ± 4 12 ± 4 57 ± 15	12 ± 4 11 ± 5 9 ± 4 10 ± 4 11 ± 4 53 ± 16	-1 (-2;1) -1 (-2;1) -1 (-3;0) -1 (-2;0) -1 (-3;0) -5 (-10;0)	12 ± 4 10 ± 4 9 ± 4 11 ± 4 12 ± 4 54 ± 18	12 ± 5 11 ± 5 10 ± 5 11 ± 5 11 ± 5 55 ± 20	0 (-1;1) 1 (-1;2) 0 (-1;1) 0 (-1;1) 0 (-1;1) 0 (-4;5)
**Anxiety and depression (HADS)**						
Anxiety Depression Sum score	7 ± 4 5 ± 4 11 ± 6	6 ± 4 4 ± 3 10 ± 6	-1 (-2;1) -1 (-2;1) -1 (-3;1)	7 ± 4 5 ± 4 12 ± 8	6 ± 4 5 ± 4 11 ± 8	0 (-1;0) 0 (-1;1) -1 (-2;1)
Return to work^A^						
No, n(%) Yes, n(%) Hours returned (%)*	20 (51%) 19 (49%) 22 ± 32	8 (24%) 25 (76%) 51 ± 43	- - 29 (16;42*)	17 (45%) 21 (55%) 23 ± 29	10 (29%) 24 (71%) 58 ± 49	- - 35 (18;51*)

Means ± SD are presented for both groups and timepoints. Mean changes over time (∆) are presented wit corresponding 95% confidence intervals (95% CI). * statistically significant

^A^ RTW was not imputed for participants who were not employed (anymore) before the diagnosis of cancer Group A *n* = 38; Group B *n* = 39)

COACH = the group of participants receiving a remote coaching intervention; CONTROL = the group of participants receiving no intervention; T0 = baseline; T1 = follow-up

EORTC-QLQ-C30 = European Organization for Research and Treatment of Cancer Quality of Life Questionnaire Core-30, MFI-20= Multidimensional Fatigue Inventory-20, HADS = Hospital Anxiety and Depression Scale

**Fig. 2:**
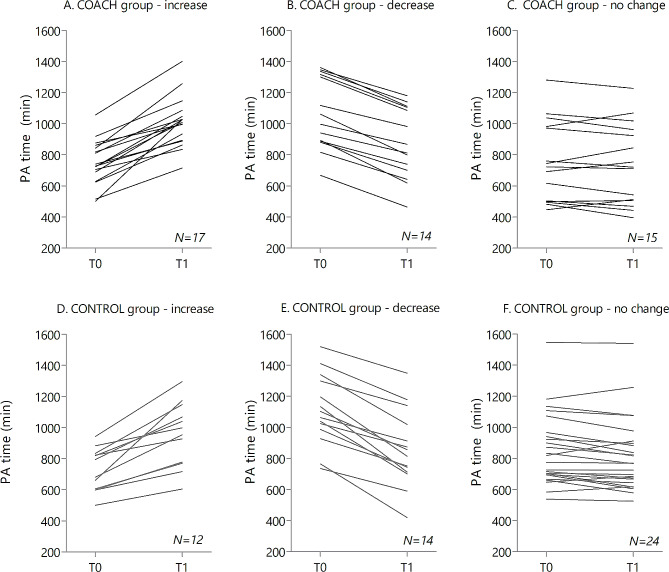
Individual participant changes from T0 to T1 in weekly accelerometer-derived physical activity (min) for participants in the COACH group who showed a relevant increase (**A**), decrease (**B**) or no change (**C**) in weekly physical activity, and for participants in the CONTROL group who showed a relevant increase (**D**), decrease (**E**) or no change (**F**) in weekly physical activity. Values of some patients are based on mean of multiple imputed values. T0 = baseline assessment, start of the study; T1 = outcome assessment, 6 months after the start of the study

In the COACH group, 17 participants (37%) showed a clinically relevant increase (≥ 105 min) in weekly accelerometer-derived PA, 15 participants (33%) remained stable and 14 participants (30%) showed a clinically relevant decrease (≤ 105 min). In the control group, 12 participants (24%) showed a clinically relevant increase (≥ 105 min), 24 participants (48%) showed no change and 14 participants (28%) showed a clinically relevant decrease (≤ 105 min) in weekly accelerometer-derived PA. Clinical relevant changes were not statistically significantly different between groups (*p* = 0.58). Individual participant changes from T0 to T1 in weekly accelerometer-derived physical activity are visualised in line graphs for both groups (Fig. 2).

### Between-group differences

After adjusting for sex, age and baseline weekly accelerometer-derived total PA, no significant between-group differences were seen at T1 for weekly accelerometer-derived total PA, VO_2_peak, 1-RM leg press, 1-RM chest press and sum scores for HRQoL, fatigue, anxiety and depression and RTW (Table 3). Ten participants in the COACH group (22%) and ten participants in the CONTROL group (20%) went to the physical therapist during the study period, so co-interventions were equally divided between groups. At T1, weekly accelerometer-derived total PA was on average 881 ± 268 min in the COACH group and 864 ± 253 min in the CONTROL group. For both groups, this was equal to 14 ± 4% of their waking time. An adjusted mean difference of 45 min (95%CI -50;140, *p* = 0.35) was seen for the COACH group minus the CONTROL group at T1, indicating slightly higher levels of PA in the COACH group, although not statistically significant. This was confirmed by the Cohen’s effect size of *d* = 0.17, indicating a small effect. Weekly sedentary time during waking hours was 4010 ± 819 min (64 ± 11%) in the COACH group and 4002 ± 765 min (63 ± 11%) in the CONTROL group, resulting in an adjusted mean difference of -36 (95%CI -389;318, *p* = 0.84) min per week. (Table 4) The COACH group reached a mean VO_2_peak of 22.3 ± 6.1 (71 ± 17% of predicted), while the CONTROL group showed a mean value of 22.7 ± 6.4 (75 ± 19% of predicted). (Table 2) At T1, 19 participants in the COACH group (41%) and 18 participants in the CONTROL group (36%) reached a VO_2_peak beneath the lower limit of normal [21]. A small, but non-significant effect was found for HRQoL as well, with a corrected mean difference of 4.0 points (CI -2.9;10.0) on the EORTC-QLQ-C30, and an effect size of *d* = 0.26 favoring the COACH group.

**Table 4:** Between group differences at T1 using linear regression^A^

	Mean difference ^B^ (95% CI)	*P*-value	Cohen’s *d* effect size^d^
Weekly accelerometer-derived total PA (min)	45 (-50;140)	0.35	**0.17**
Weekly accelerometer-derived sedentary wake time (min) ^C^	-36 (-389;318)	0.84	**-0.05**
Weekly self-reported weekly MVPA (min)	-16 (-190;158)	0.86	**-0.04**
Peak oxygen uptake, VO_2_ peak (mL/kg/min)	-0.7 (-2.9;1.4)	0.50	**-0.11**
1-RM leg press (kg)	2(-12;17)	0.77	**0.06**
1-RM chest press (kg)	0 (-4;5)	0.94	**0.00**
Quality of Life, EORTC-QLQ- C30 Sum score	4.0 (-2.9;10.0)	0.28	**0.26**
Fatigue, MFI Sum score	-2 (-10;6)	0.52	**-0.11**
Anxiety and depression, HADS Sum score	-1 (-4;2)	0.42	**-0.14**
Return to work, percentage hours returned (%)	-7 (-29;16)	0.55	**-0.15**

^A^ Corrected for gender, age and baseline values of weekly physical activity

^B^ Mean difference is = unstandardised B; COACH group minus CONTROL group

^C^ Weekly time that participants were sedentary during waking hours

^d^ Calculated as the corrected mean difference divided by the pooled standard deviation for both groups at T1

T1 = follow-up; PA = physical activity, MVPA = moderate-to-vigorous physical activity, VO2peak = peak oxygen uptake 1-RM = one-repetition maximum EORTC-QLQ-C30 = European Organization for Research and Treatment of Cancer Quality of Life Questionnaire Core-30, MFI-20= Multidimensional Fatigue Inventory-20, HADS = Hospital Anxiety and Depression Scale

## Discussion

This study shows that extending a supervised exercise-based oncology rehabilitation program with our six-month remote coaching intervention had no significant benefits compared to no additional intervention. No significant between-group differences were seen for weekly accelerometer-derived PA levels, sedentary time and self-reported MVPA levels, aerobic capacity, upper and lower body muscle strength, quality of life, fatigue, anxiety and depression and RTW, after six months of receiving or not receiving a remote coaching intervention. An adjusted mean difference in weekly accelerometer-derived total PA of 45 min was seen between the COACH group and the CONTROL group at T1, favouring the COACH group, and a small effect size of *d* = 0.17, but effects were not statistically nor clinically relevant [28]. Return to work increased significantly in both groups, while all other outcomes remained stable within both groups, six months after completing the supervised exercise program. However, non-significant within-group changes of + 33 min in the COACH group and − 30 min in the CONTROL group were seen in the primary outcome measure accelerometer-derived PA.

We hypothesised that the COACH group would maintain or improve PA levels, while the CONTROL group would show a decrease. However, results showed that 70% and 72% of the participants in the COACH group and CONTROL group respectively, were able to maintain or improve PA levels six months after completing supervised exercise oncology rehabilitation. No significant between-group differences were seen for the distribution of participants that showed a decrease, an increase, or no change in PA levels. The ability to maintain PA levels after a supervised rehabilitation program varied considerably across participants and was not affected by a remote coaching intervention. (Fig. 2)

At T1, participants in the COACH group had a total accelerometer-derived PA of 881 ± 268 min/week compared to 864 ± 253 min/week in the CONTROL group. For both groups, this was equal to 14 ± 4% of their waking time. Participants in the COACH group and the CONTROL group spent on average 64% and 63% of their waking time sedentary. The PA levels in the current study are comparable, but slightly higher compared to those of patients with colorectal cancer in a Dutch cohort study (*n* = 114), who were older than our population (mean age 70.0 years) and showed a physical activity time of 1.7 h/day, equal to 714 min per week, measured with the MOX accelerometer [33]. In an RCT by O’Neill et al., a higher mean total PA time of 1650 min/week was found in participants with esophagogastric cancer in Ireland (*n* = 22, mean age 61.4 years), measured with the ActiGraph accelerometer, 3 months after participating in a 12-week multidisciplinary rehabilitation program containing supervised exercise, with no significant changes over time since the end of the program [34]. Sweegers et al. [35] pooled ActiGraph accelerometer data of 1447 cancer survivors from the Netherlands, Australia, Canada and the United states, with a mean age of 59.3 years and a median time since medical treatment of 46.6 months. They reported that participants spent on average 66% of their day sedentary, which is in accordance with the results of the current study. Total physical activity, on the other hand, was much higher in their study, with 297 min/day, or 2075 min/week. This discrepancy could be partly explained by the fact that time spent in standing posture was included in PA time in their study, while this was not the case in the current study. Large differences in PA time between studies could be due to differences in the population (e.g. age, diagnosis, living area) and the use of different accelerometers. Besides, in some of the studies participants took part in a rehabilitation program, while this was not the case in other studies.

It is difficult to further interpret the values for weekly total PA time correctly, because normative values or guidelines do not exist. The WHO guideline only reports thresholds on the recommended amount of minutes/week spent in MVPA (PA with an intensity ≥ 3.0METs) [36]. In this study, we did not subdivide PA, because of a limited reproducibility of the MOX-accelerometer for estimating minutes of MVPA [17]. While the recent guidelines only report thresholds on the amount of MVPA per week, the recommendation to minimize sedentary behavior was added [36]. This was underpinned with the acknowledgement that replacing sedentary time with any intensity of PA (including light activity), has health benefits. However, there is still insufficient evidence to determine quantitative thresholds and specific recommendations on reducing sedentary behavior apart from MVPA. We asked participants to keep a PA diary in order to get insight in minutes of MVPA. At T1 participants in the COACH group reported 557 ± 400 min of MVPA, while the CONTROL group reported 589 ± 414 min. These values are much higher than the WHO guidelines of 150–300 min. It can be expected that time of MVPA was highly overestimated by the participants, as was concluded by Smith et al. in a study about self-reported PA in patients with prostate cancer [37]. 

In contrast to the findings of our study, a meta-analysis of Roberts et al. showed significant positive effects for digital interventions on PA levels in cancer survivors (mean difference in MVPA = 49 min/week, 95% CI 16; 82). However, the included studies used self-reported PA as outcomes and high levels of heterogeneity were seen [38]. Gomersal et al. reported that a 12-week tailored text messaging intervention, additional to a standard-care 4-week oncology rehabilitation program had beneficial effects on sitting time and time spent in light-intensity PA, but not on MVPA, measured with the activPAL accelerometer [10]. In a study of Gell et al., cancer survivors who received tailored advice from a health coach and follow-up phone calls and messages, combined with a Fitbit activity monitor for goal setting following an exercise-based rehabilitation program maintained accelerometer-derived (Actigraph) MVPA levels eight weeks later, while participants who got a Fitbit activity monitor with one-off advice only, showed a significant decline in MVPA minutes [9]. 

In the current study, we also assessed aerobic capacity. Results showed that aerobic capacity remained stable from T0 to T1 in both groups, without between-group differences. At T1, participants in the COACH group reached mean a VO_2_peak of 22.2 mL/kg/min (71% of predicted), while participants in the CONTROL group had a mean VO_2_peak of 22.6 mL/kg/min (74% of predicted). For 41% and 36% of the participants in the COACH group and the CONTROL group respectively, these values were below the lower limit of normal [21]. These findings confirm that a 10-week supervised rehabilitation program was not sufficient to reach normal levels of aerobic capacity and, in contrast to our hypothesis, additional remote coaching had no beneficial effects. This is worrying, since aerobic capacity can be seen as a clinical vital sign and is inversely related to all-cause and cancer-related mortality [39, 40]. 

Contrary to our expectations and findings of previous studies, our remote coaching intervention following supervised exercise rehabilitation did not show to be significantly effective to improve PA levels, sedentary time, performance-based and patient-reported outcomes. One potential explanation is the fact that a relatively motivated group of participants was selected for this study, since they were willing to attend the supervised rehabilitation program in the first place and consented to participate in this study afterwards. These patients might have been more motivated to sustain or increase PA levels, compared to the general population of cancer survivors. This was confirmed by our data, showing that 72% of the participants in the CONTROL group, who did not receive any additional intervention after the supervised rehabilitation, was able to maintain or increase PA levels. Moreover, participants in this study were relatively young compared to the general cancer population. The mean age was comparable to other studies on exercise oncology rehabilitation, which indicates that more research is needed on targeting older cancer survivors for oncology rehabilitation. [13, 41] Potentially, the effects of remote coaching investigated in this study would have been significant if only patients in need were targeted. Harris et al. described that elderly participating in a physical activity study reported greater physical activity than the non-participants [42]. Furthermore, the study information and the follow-up measurements may have been a stimulus for participants to sustain PA levels. Receiving information about the study might have raised the awareness for PA maintenance and the prospect of follow-up measurements potentially motivated people to stay active. Besides, participants may have increased PA during the week of the accelerometer measurement. This phenomenon is known as measurement reactivity, meaning that behavior is likely to change when it is monitored [43]. However, this probably occurred in both the COACH group and the CONTROL group and did therefore not influence intervention effects. This can be confirmed by the finding that aerobic capacity remained stable over time and did not differ between groups either, since increasing PA in the week of the measurement does not influence outcomes of aerobic capacity.

### Strengths, limitations and future recommendations

Strengths of our study included the objective and accurate measurement of PA and sedentary behavior using the MOX accelerometer and aerobic capacity using the CPET. However, more research is needed to determine thresholds for categorising intensities of PA using objective PA measurements, such as accelerometry. Furthermore, a broad spectrum of variables was collected, covering not only physical but also psychosocial outcomes and fatigue. One of the limitations was the fact that participants were recruited from a multidisciplinary rehabilitation program, suitable for patients who experience both physical and psychosocial complaints and/or chronic fatigue. Therefore, the findings of this study are not generalisable to all cancer survivors. Besides, PA behaviour might have changed during the course of this study because of the COVID-19 pandemic. However, because of the randomised controlled design, this is unlikely to have distorted the study results. The majority of the participants completed the intervention as intended despite the COVID-19 measures. Another limitation was the fact that intervention dose and duration were equal for all participants in this study. This intervention should be optimised and personalised in the future. Important keys that play a role in PA maintenance should be taken into account when optimizing the intervention. A qualitative study showed that the remote coaching intervention investigated in the current study was acceptable for cancer survivors, but added value differed between patients. For some participants, the intervention could be improved by adding face-to-face appointments. Self-efficacy, accountability, PA habits, physical complaints and accessibility of facilities were key themes for PA maintenance and should therefore be taken into consideration when improving the intervention. [44]

Future research should focus on identifying determinants (e.g. patient characteristics, medical status, social environment) that are related to PA maintenance after supervised rehabilitation. This would enable healthcare providers to monitor the patients at risk beyond the program and offer them a follow-up intervention. In addition, the content of remote coaching could be improved accordingly and tested for efficacy. Since reaching, and motivating patients through remote coaching interventions is challenging, appropriate methods to achieve this should be explored, as well as the acceptability of these interventions in the target population. Another limitation, is the fact that little is known about the minimal clinically important change in PA. Future research should look into the minimal change in PA that is relevant for cancer survivors. Lastly, after optimising this remote coaching intervention, knowledge should be expanded to adjacent regions and effectiveness should be investigated at a larger scale.

## Conclusion

Extending a supervised exercise oncology rehabilitation program with a six-month remote coaching intervention was not effective to improve maintenance of PA levels; aerobic capacity; muscle strength; and patient-reported outcomes in cancer survivors. However, a non-significant mean difference of 45 min in PA was found, favouring the group of participants that received the remote coaching intervention. More research is needed to identify patients most in need for follow-up interventions following supervised exercise program and to investigate the effectiveness of remote coaching interventions in these patients.

### Electronic supplementary material

Below is the link to the electronic supplementary material.

Supplementary Material 1

Supplementary Material 2

Supplementary Material 3

## Data Availability

Data are available upon reasonable request.

## Abbreviations

COACH group: The group of participants receiving a remote coaching intervention
COM-B model: Capability, Opportunity and Motivation model of Behaviour
CONSORT: Consolidated Standards of Reporting Trials
CONTROL group: The group of participants receiving no intervention
COVID-19: Coronavirus-19
CPET: Cardiopulmonary exercise test
EORTC QLQ-C30: European Organization for Research and Treatment of Cancer Quality of Life Questionnaire Core-30
HADS: Hospital Anxiety and Depression Scale
HRQoL: Health-Related Quality of Life
ICC: Intraclass correlation coefficient
MET: Metabolic Equivalent of Task
MFI-20: Multidimensional Fatigue Inventory-20
MUMC +: Maastricht University Medical Centre +
MVPA: Moderate-to-vigorous intensity physical activity
OUES: Oxygen uptake efficiency slope
PA: Physical activity
RERpeak: Respiratory exchange rate at peak exercise
RM: Repetition maximum
rpm: Rotations per minute
RTW: Return to work
sd: Standard deviation
T0: Baseline assessment, start of the study
T1: Outcome assessment, 6 months after the start of the study
TIDieR: Template for intervention Description and Replication
VO_2_: Oxygen uptake
VO_2_peak: Peak oxygen uptake
VO_2_RCP: Oxygen uptake at the respiratory compensation point
VO_2_VAT: Oxygen uptake at the ventilatory anaerobic treshold
WHO: World Health Organization
95% CI: 95% confidence intervals
