# Supplementary material for: Effects of remote coaching following supervised exercise oncology rehabilitation on physical activity levels, physical fitness, and patient-reported outcomes: a randomised controlled trial
Source: Int J Behav Nutr Phys Act. 2024 Jan 25;21:8. doi: 10.1186/s12966-024-01561-2 (PMC10809633; doi:10.1186/s12966-024-01561-2)
Supplement: Supplementary file 2 — Supplementary Material 2 [file 12966_2024_1561_MOESM2_ESM.docx]

| **Additional file 2.\|** **Intake questionnaire remote coaching intervention according to the COM-B model.**  *Anouk T.R. Weemaes, MSc, PT^1,2^(ORCID 0000-0003-4611-6626), Milou Beelen, PhD, MD^1,3^ (ORCID: 0000-0002-0065-1167), Prof. Matty P. Weijenberg ,PhD^4^ (ORCID 0000-0003-1695-4768), Dr. Sander M. J. van Kuijk, PhD^5^(ORCID 0000-0003-2796-729X), Prof. Antoine F. Lenssen, PhD, PT^1,2^ (ORCID: 0000-0003-3627-4452)* | | |
| --- | --- | --- |
| Physical capacity | I spend at least 30 minutes daily performing physical activities that make my heartrate and breathing frequency raise. (e.g. brisk walking, cycling, swimming, running fitness, ball games). | YES 52% NO 48% |
| Psychological capacity | I know what the recommended level of physical activity is according to current guideline. | YES 95% NO 5% |
|  | I know what kinds of physical activities are suitable for improving my health. | YES 31% NO 69% |
|  | I know what kinds of physical activities are not suitable for improving my health (e.g. because of injuries or health issues). | YES 93% NO 7% |
|  | I talk regularly (at least once a week) about the theme physical activity with others. | YES 75% NO 25% |
|  | I know how to make a good planning, in order to perform sufficient physical activity. | YES 43% NO 57% |
|  | I know how to monitor my physical activity. | YES 75% NO 25% |
|  | I know how to avoid skipping planned physical activities. | YES 89% NO 11% |
| Reflective motivation | I would identify myself as someone who finds it important to be sufficiently active. | YES 75% NO 25% |
|  | I am responsible for performing sufficient physical activity. | YES 100% NO 0% |
|  | I am confident that I will manage to perform sufficient physical activity. | YES 100% NO 0% |
|  | I believe that having an active lifestyle is good for my health. | YES 86% NO 14% |
|  | I believe that having an active lifestyle has a positive effects on my working- and private environment. | YES 93% NO 7% |
|  | I believe that performing sufficient physical activity makes people feel good. | YES 100% NO 0% |
|  | I believe that performing insufficient physical activity makes people feel guilty. | YES 100% NO 0% |
|  | I have decided to start performing sufficient physical activity. | YES 80% NO 20% |
|  | It takes little effort for me to perform sufficient physical activity. | YES 50% NO 50% |
|  | I am willing to give up other activities, in order to perform sufficient physical activity. | YES 77% NO 23% |
|  | I have clearly in mind what I want to achieve during the remote coaching intervention. | YES 91% NO 9% |
| Automatic motivation | I encourage myself to make performing sufficient physical activity a habit. | YES 89% NO 11% |
|  | Performing sufficient physical activity makes me feel good. | YES 95% NO 5% |
| Physical opportunity | I feel like I have the resources in my environment (e.g., time, money, transportation, materials needed) to perform sufficient physical activity. | YES 80% NO 20% |
|  | I avoid situations or stimuli in my environment that prevent me from performing sufficient physical activity. | YES 43% NO 57% |
|  | There are cues or reminders in my environment that remind me to perform sufficient physical activity. | YES 64% NO 36% |
| Social opportunity | I believe that most of the people I hang out with perform sufficient physical activity. | YES 64% NO 36% |
|  | I believe that most of the people I hang out with believe I should perform enough physical activity. | YES 73% NO 27% |
|  | I feel like I have enough social support to be perform sufficient physical activity. | YES 84% NO 16% |
